# Multitracer Stable Isotope Quantification of Arginase and Nitric Oxide Synthase Activity in a Mouse Model of Pseudomonas Lung Infection

**DOI:** 10.1155/2014/323526

**Published:** 2014-08-11

**Authors:** Hartmut Grasemann, Thomas Jaecklin, Anne Mehl, Hailu Huang, Mahroukh Rafii, Paul Pencharz, Felix Ratjen

**Affiliations:** ^1^Program in Physiology and Experimental Medicine, SickKids Research Institute and Division of Respiratory Medicine, The Hospital for Sick Children, University of Toronto, 555 University Avenue, Toronto, ON, Canada M5G 1X8; ^2^Department of Pediatrics, The Hospital for Sick Children, University of Toronto, 555 University Avenue, Toronto, ON, Canada M5G 1X8; ^3^Division of Critical Care Medicine, University of Toronto, 555 University Avenue, Toronto, ON, Canada M5G 1X8

## Abstract

Cystic fibrosis airways are deficient for L-arginine, a substrate for nitric oxide synthases (NOSs) and arginases. The rationale for this study was to quantify NOS and arginase activity in the mouse lung. Anesthetized unventilated mice received a primed constant stable isotope intravenous infusion containing labeled L-arginine, ornithine, and citrulline. The isotopic enrichment of each of the infused isotopomers and its product amino acids were measured in plasma and organ homogenates using liquid chromatography-tandem mass spectrometry. The effect of infection was studied three days after direct tracheal instillation of Pseudomonas-coated agar beads. In the infusion model, lung infection resulted in a significant (28-fold) increase in NOS activity in lung but not in trachea, kidney, liver, or plasma. Absolute rates of arginase activity in solid tissues could not be calculated in this model. In an isolated lung perfusion model used for comparison increased NOS activity in infected lungs was confirmed (28.5-fold) and lung arginase activity was increased 9.7-fold. The activity of L-arginine metabolizing enzymes can be measured using stable isotope conversion in the mouse. Accumulation of L-ornithine in the whole mouse model hindered the exact quantification of arginase activity in the lung, a problem that was overcome utilizing an isolated lung perfusion model.

## 1. Introduction

Nitric oxide synthases (NOS) and arginases compete for the amino acid L-arginine as substrate. The isoforms of NOS produce nitric oxide (NO) and L-citrulline, whereas arginases produce urea and L-ornithine [1]. The known interactions between the two pathways are thought to result in reciprocal regulation of the* in vivo* enzyme activities. For instance, N^*ω*^-hydroxy-L-arginine (NOHA), formed as an intermediate during NO production from NOS, acts as an arginase inhibitor and arginase activity also decreases with S-nitrosylation of the enzyme [1]. Increased arginase activity can cause substrate limitation for and uncoupling of NOS, leading to reduced NO and a switch to superoxide and subsequently peroxynitrite production [2]. NOS can also be affected by products of arginase activity, as the L-ornithine derived polyamines act as NOS inhibitors [3]. Other endogenous NOS inhibitors include methylated arginine derivates such as the asymmetric dimethylarginine (ADMA) [1–6].

Increased NOS expression has been shown to occur in infection or inflammation, but quantification of NOS activity in specific organs or tissues is usually indirect often by measuring NO metabolite concentrations. However, nitrate and nitrite concentrations may not accurately reflect tissue NOS activity [7, 8] specifically in the presence of denitrifying bacteria [9]. Multitracer stable isotopes have previously been used to measure systemic L-arginine metabolism in humans and in animals [10–12]. We here aimed to develop methods using stable isotopes in the mouse that would allow quantification of whole body and organ-specific activities of the L-arginine metabolizing enzymes arginase and nitric oxide synthase and changes in activity of these enzymes in response to* Pseudomonas aeruginosa* infection of the lung.

## 2. Methods

The experiments were approved by the institutional Animal Care Committee and were conducted in accordance with the guidelines of the Canadian Council for Animal Care.

### 2.1. Whole Mouse Infusion Model

A detailed description of the multitracer studies and analytic procedures can be found in the supplement (see Supplementary Material available online at http://dx.doi.org/10.1155/2014/323526). A stable isotope solution containing L-arginine (m+6 L-arginine U-^13^C_6_), L-ornithine (m+2 L-ornithine ^15^N_2_), and L-citrulline (m+5 L-citrulline 5-^13^C; 4,4,5,5-H_4_) in normal saline (0.9%) was used similarly as previously described [10]. Pilot experiments using each of the three isotopes individually and in combinations were performed to demonstrate no interaction of the isotopes when used in the final combination. Primed constant isotope infusion with the final combination for a length of up to 60 min resulted in isotopic steady state after 30 min (see Figure 1 in supplement). In the actual experiments, infusions were delivered beginning with a bolus given within 20 seconds, followed by a constant infusion of the isotope mix at 1 mL/hr for 45 minutes (see Table 1).

To administer the infusion into the living mouse, a small incision was made in the neck of the animal and a jugular vein dissected. A 30-gauge tubing (Tygon Micro-Bore tubing, Saint Gobain Performance Plastics) was inserted into the vein through a small incision and fixated with help of a ligature thread. The distal end of the tubing was connected to a needle attached to a syringe that contained the isotope solution. The infusion was delivered by a single-syringe infusion pump (KDS 100, kdScientific, Holliston, MA) at a rate of 1 mL/hr for 45 min, while the mice were situated on a mouse warming pad at 37°C. Following the infusion, mice were euthanized with ketamine/xylazine and necropsy was performed. Isolated serum and lung, kidneys, and liver were snap-frozen in liquid nitrogen and then stored at −80°C.

### 2.2. Isolated Lung Perfusion Model

A previously described isolated lung perfusion model [13] was utilized to study L-arginine metabolism in the lung* ex vivo*. Lungs were ventilated with negative pressure ventilation (end-expiratory pressure -3 cm H_2_O, tidal volume 10 ± 2 mL/kg, rate 92/min, and gas mixture of 5% CO_2_, 30% O_2, _and balanced N_2_) and perfused (1 mL/min constant rate) with a blood-free perfusate containing physiological but labeled concentrations of L-arginine (m+6, 150 *µ*M), L-ornithine (m+2, 134 *µ*M), and L-citrulline (m+5, 90 *µ*M) stable isotopes. The osmolality of the perfusate was adjusted to 340 mmol/L by adding NaCl. The perfusion circuit, reservoir, and mouse lungs were kept at 37°C during the experiment. The perfusion was administered in a single-pass of fresh solution and perfusate recovered from the lungs was discharged. Lungs were harvested after 45 min and stored at −80°C.

### 2.3. Liquid Chromatography-Tandem Mass Spectrometry and Calculations

Each of the infused isotopomers and its product amino acids were measured in serum and organ tissue homogenates using liquid chromatography-tandem mass spectrometry, similar to what is previously described [10, 11]. The isotopic enrichment was determined using the previously described formulas [10, 14]. Enrichment of the product of enzymatic conversion (transfer of label from precursor to product) was expressed as moles percent excess (MPE). Absolute conversion rates were calculated as the fractional conversion (enrichment of product divided by enrichment of the precursor) multiplied by the flux rate of the product (see supplement). Logically, the enrichment of the product must be equal to or less than the enrichment of the precursor; that is, the MPE ratio (or fractional conversion) should be ≤1, unless there is delayed clearance of the product from the tissue. This, however, appeared to be the case for arginase (i.e., enrichment of ornithine derived from arginine) in solid tissues of living mice in the infusion model. Tissue arginase activity in these studies is therefore expressed as product enrichment.

### 2.4. Mice and Infection Protocol

Eight-to-ten-week-old female C57BL/6 mice purchased from Charles River Laboratories (Charles River, Oakville, Quebec, Canada) were housed in a pathogen-free environment and received autoclaved food and water in the laboratory animal services at our institution.

Agarose beads embedded with* Pseudomonas aeruginosa* (mPAO1) were made following a published protocol [15] and modified by us. Briefly, bacteria were grown overnight in trypticase soy broth (TSB, Fisher scientific) at 37°C and 2% agar in phosphate buffered saline (PBS), pH 7.4, was mixed with the bacteria broth when bacteria were in late log phase. The agar broth mixture was added to heavy mineral oil that was equilibrated at 50–55°C, rapidly stirred for 6 min at room temperature, and then cooled over 10 min. The beads were washed with 0.5% and 0.25% sodium deoxycholate in PBS and then washed 3-4 times with PBS. Larger beads were removed by using a spectra mesh filter (Opening Ø213 M, Spectrum Laboratories); thereafter, more than 80% of beads are between 70 and 200 *µ*m. Finally, 10-fold serial dilutions of a homogenized aliquot of the bead slurry were platted on Trypticase Soy Agar (TSA, Becton Dickinson Company) and incubated at 37°C for 18–20h. Bacterial colony counts were then recorded as colony-forming units (CFU)/mL. Sterile control beads were prepared identically except bacteria that were absent and confirmed to be sterile by checking for bacterial growth after plating a subsample on agar before each use.

Beads were injected into the airways after intubation under direct vision as previously described [16] in anaesthetized mice (ketamine 150 mg/kg and xylazine 10 mg/kg administered intraperitoneally). A final* P. aeruginosa* dose of 2 × 10^6^ CFU in a volume of 40–50 *µ*L was injected into the trachea. Body weight was monitored daily, prior to, and for 3 days following the infection. Mice underwent the above described stable isotope studies on day three of the infection.

Arginase activity was also measured by conversion of L-arginine to ornithine* in vitro*, as previously described [17] and modified by us [18, 19]. The NO metabolites nitrate and nitrite were measured in lung tissue homogenate using the Griess reagent, as previously described [20]. NO metabolite (nitrate + nitrite) concentrations were expressed as nmol/mg protein. Immunoblotting was performed as previously described, and bands on imaging film were quantified by densitometry and expressed as a ratio to the corresponding GAPDH densities [18, 21]. Antibodies for immunoblotting were purchased from Santa Cruz Biotechnology (Dallas, TX).

Data were expressed as mean ± SEM. Group comparisons were made by *t*-test or Mann-Whitney test, where appropriate. A *P* value of <0.05 was considered significant. Analysis of variance (ANOVA) was used for repeated measures of body weight.

## 3. Results

### 3.1. Whole Mouse Infusion Mouse Model

The plasma MPE ratios in naïve mice (*n* = 6) were 0.015 ± 0.001 for NOS (MPE Arg > Cit/MPE Arg) and 1.05 ± 0.12 for arginase (MPE Arg > Orn/MPE Arg). Corresponding calculated plasma enzyme activities were 1.46 ± 0.19 nmol/g mouse/h for NOS and 288.1 ± 63.5 nmol/g mouse/h for arginase. In solid tissues, MPE ratios for NOS were also <1 in naïve mice. Calculated NOS activities for lung, trachea, liver, and kidney are shown in Table 2. Unlike for NOS, ornithine enrichment from arginine exceeded labeled arginine MPE in all solid tissues studied (lung, trachea, kidney, and liver), which resulted in arginase MPE ratios of greater >1, respectively. As accurate calculations of enzyme activities are imprecise using MPE ratios that largely exceed 1, we therefore expressed solid tissues arginase activity in the whole mouse infusion model as ornithine enrichment from arginine (or MPE Arg > Orn) (Table 2).

**Figure 1:**
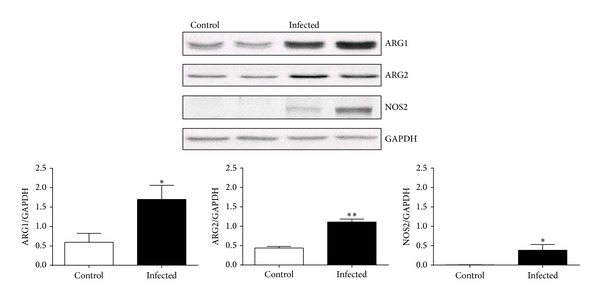
Expression of inducible nitric oxide synthase (NOS2) and the arginase isoform types I (ARG1) and II (ARG2) normalized to GAPDH, respectively, in lung of* Pseudomonas aeruginosa* infected mice and naïve control (*n* = 6 per group). Infection resulted in a significant increase in protein expression of all three enzymes (*P < 0.05, **P < 0.0001).

*Pseudomonas* infection was associated with significant weight loss and a 14% reduction in body weight on day 3 (19.2 ± 0.5 versus 16.5 ± 0.4 g, *P* < 0.001, ANOVA) of the infection. No change in weight was observed in the noninfected control animals (20.6 ± 0.8 versus 20.9 ± 0.7 g). As expected, infection resulted in a significant increase not only in protein expression of NOS 2 (0.384 ± 0.149 versus 0.006 ± 0.0016 NOS2/GAPDH, *P* = 0.029), but also in arginase 1 (1.97 ± 0.29 versus 0.71 ± 0.24 Arg 1/GAPDH, *P* = 0.01) and arginase 2 (1.11 ± 0.08 versus 0.44 ± 0.04 Arg 2/GAPDH, *P* < 0.0001) in lung (*n* = 6 per group) (Figure 1).

**Figure 2:**
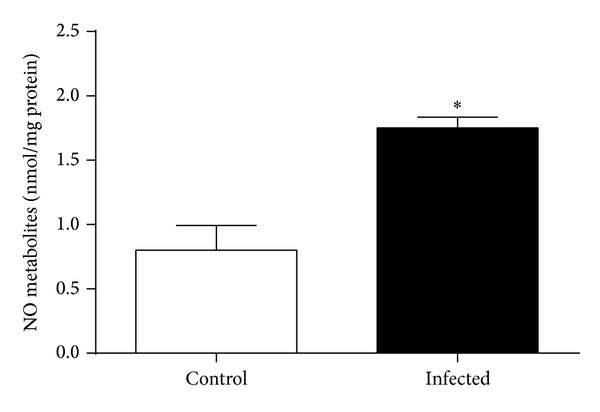
NO metabolite (nitrate+nitrite) concentrations in lung homogenates of mice infected with* Pseudomonas aeruginosa* and noninfected controls (*n* = 6 per group). NO metabolite concentrations were higher in the infected animals (*P < 0.001).

**Figure 3:**
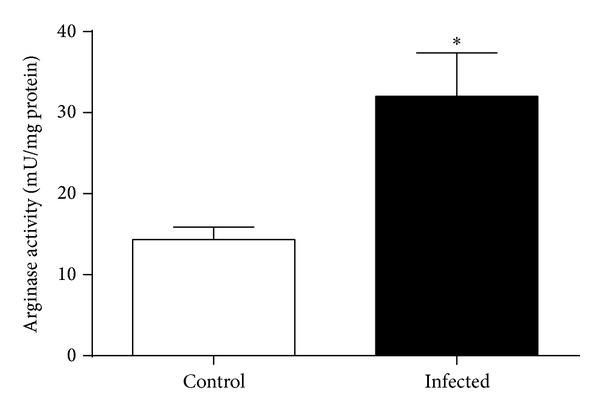
Arginase activity measured in lung homogenates of mice infected with* Pseudomonas aeruginosa* and noninfected controls* in vitro* (*n* = 4 per group) (*P < 0.05).

NO metabolite (nitrate + nitrite) concentrations were significantly increased in lung homogenates of* Pseudomonas* infected mice compared to controls (1.75 ± 0.09 versus 0.80 ± 0.19 nmol/mg protein, *P* < 0.001) (*n* = 6 per group) (Figure 2), as was* in vitro* arginase activity (32.0 ± 5.4 versus 14.3 ± 1.6 mU/mg protein, *P* = 0.028) (*n* = 4 per group) (Figure 3).

Enzyme activities measured after stable isotope infusion in* Pseudomonas* infected mice demonstrated mean plasma MPE ratios of 0.025 ± 0.003 for NOS and 0.87 ± 0.05 for arginase (*n* = 7). Calculated plasma enzyme activities after infection were 1.63 ± 0.2 nmol/g mouse/h for NOS and 242.5 ± 95.7 nmol/g mouse/h for arginase, which was not significantly different from the naïve controls (*P* = 0.579 and *P* = 0.709, resp.). Calculated tissue NOS activity in infected animals was significantly increased in lung but not in trachea, liver, or kidney (Table 2). In contrast, arginase MPE in infected animals showed a small but statistically significant increase for lung, trachea, and liver, but not for kidney (Table 2).

### 3.2. Isolated Lung Perfusion Model

**Figure 4:**
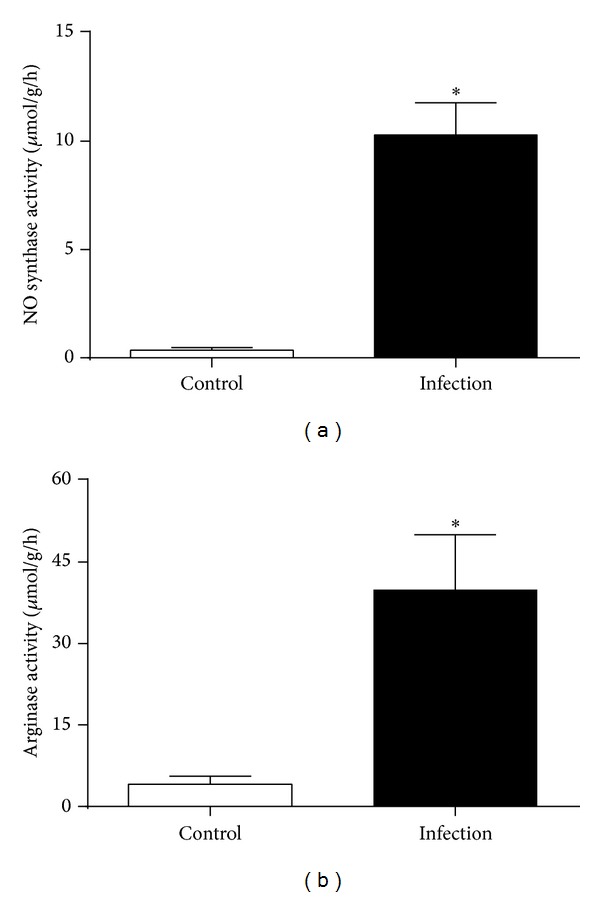
(a) Nitric oxide synthase (NOS) and (b) arginase activity in mouse lung calculated from stable isotope conversion in an isolated lung perfusion model. NOS and arginase activity were significantly increased three days after direct tracheal instillation of* Pseudomonas*-laden agarose beads (infection) compared to control (*P < 0.001, resp.).

An isolated lung perfusion model was used to confirm the findings from the whole mouse infusion model. In the isolated lung perfusion model, the mean MPE ratio in naïve mice (*n* = 5) was 0.037 ± 0.002 for NOS and 0.634 ± 0.046 for arginase. The ratio increased to 0.356 ± 0.02  (*P* < 0.0001) for NOS in infected animals (*n* = 5). For arginase, the ratio was ≤1 in 4 out of 5 lungs and the mean was 1.183 ± 0.18  (*P* = 0.0184). Calculated NOS activity in lung increased from 0.36 ± 0.12 to 10.30 ± 1.48 *µ*mol/g lung/h (28.5-fold) and arginase activity increased from 4.12 ± 1.49 to 39.9 ± 10.2 *µ*mol/g lung/h (9.7-fold) (Figure 4).

## 4. Discussion

Using multitracer stable isotope techniques, we measured organ-specific L-arginine metabolism by NOS and arginase in the mouse* in vivo*. Infection of the lung with* Pseudomonas*-coated beads resulted in a significant increase in NOS activity in the lung but not in other organs. In contrast, arginase activity after lung infection was increased not only in lung, but also in trachea and liver.

**Figure 5:**
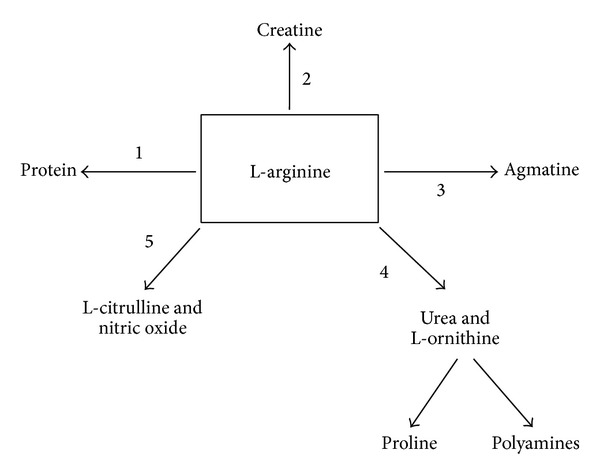
The arginine metabolism. Enzymes that use L-arginine as substrate are (1) arginyl-tRNA synthetase, (2) arginine:glycine amidinotransferase, (3) arginine decarboxylase, (4) arginases, and (5) NO synthases (NOS).

The amino acid L-arginine is substrate to five groups of enzymes in mammalians (Figure 5), which include arginyl-tRNA synthetase, arginine:glycine amidinotransferase, arginine decarboxylase, arginase, and nitric oxide synthase isoforms [22]. We here aimed to quantify the activity of arginase and NOS in the lung using stable isotopes. Similar approaches have previously been used to characterize systemic L-arginine metabolism in animals and human subjects [10–12, 23–26]. To characterize NOS and arginase activity in the whole mouse (using plasma) but also in isolated solid organs, we modified a method that was recently used by members of our group to quantify the effects of arginine intake on whole body arginine metabolism in neonatal piglets. Using primed constant intravenous infusion of stable isotopes in living mice, quantification of NOS activity was unproblematic for whole body (systemic) activity as well as for all solid organs analyzed separately (lung, trachea, liver, and kidney). In contrast, the MPE for arginase (enrichment of ornithine from arginine) was greater than the precursor MPE arginine in solid tissues (but not in plasma) at the time point of organ harvest (45 minutes of constant infusion). While this observation may be explained by differences in the kinetics of L-arginine and L-ornithine metabolism in tissue, ratios largely exceeding 1 cannot be used to calculate absolute rates of arginase activity. We therefore used the ornithine enrichment to quantify solid tissue arginase activity in this model. However, whether the ornithine enrichment is an accurate reflection of arginase activity remains unclear.

Because of this limitation, we then utilized an* ex vivo* isolated lung perfusion model using stable isotopes in the perfusate. Different from the whole mouse infusion experiments, in this model the MPE ratios for NOS and arginase were below 1 (with the exception of arginase MPE ratio in one animal), which allowed for calculation of lung enzyme activities. Increased lung NOS and arginase activities after infection of the lung with* Pseudomonas* were confirmed in this model. Both models revealed similar increases in NOS activity following infection (28-fold in the whole mouse infusion model and 28.5-fold in the isolated lung perfusion model). Infection-induced changes in lung arginase activity were 9.7-fold in the isolated lung perfusion model suggesting that arginase MPE derived from the whole mouse infusion model underestimates true arginase activity, as the change seen in the infusion model was only 1.5 fold. Interestingly, however, changes of arginase activity in an* in vitro* enzyme activity test using excess substrate concentrations in the millimolar range were more consistent with the MPE data derived from the whole animal infusion model (2.2-fold change) suggesting that this enzymatic assay is also not reflective of true arginase activity in tissue.

One potentially important difference between infusions in intact mice and the isolated lung perfusion model was that in the latter a blood-free perfusate and an open circuit were used, which means that the perfusate recovered from the lungs was not recycled. This may potentially affect tissue concentrations of factors important for enzyme activity including cofactors, endogenous inhibitors, and amino acid concentrations that determine uptake of L-arginine via CAT and thus L-arginine availability for intracellular enzymes. Nevertheless, fold changes in lung NOS activity after the infection were very similar comparing both models. In contrast, NO metabolite concentrations in lung homogenates only doubled following infection, but NO-metabolite concentration may not represent an accurate reflection of NOS* in vivo* activity [7, 8].

One strength of the described isolated organ perfusion model is that it seems to allow for an accurate calculation of both NOS and arginase and thus it can be utilized to assess the arginase/NOS activity ratio as a measure of balance of L-arginine metabolizing enzymes. Under baseline conditions L-arginine consumption by arginase exceeded that by NOS in plasma and in the lung. Interestingly, however, while infection did not significantly change the arginase/NOS activity ratio in plasma, there was evidence for significant arginase/NOS imbalance in lung as the activity ratio decreased from 11.4 in naïve to 3.9 following the infection. One limitation of our approach is that we quantified the L-arginine metabolism by arginase and NOS only, but L-arginine is involved in many other metabolic pathways (Figure 5). A characterization of the balance between L-arginine metabolizing enzymes beyond arginase and NOS may be helpful in future preclinical studies aiming to assess efficacy of therapeutic interventions targeting L-arginine homeostasis (such as arginase inhibitors), for instance, in animal models of cystic fibrosis (CF) lung infection [27]. Studies in CF have previously demonstrated increased arginase activity, increased concentrations of the endogenous NOS inhibitors asymmetric dimethylarginine (ADMA) and spermine, reduced L-arginine availability for NOS, and reduced NO formation in CF airways [19, 28–32].

In summary, our results support previous work that systemic and organ-specific arginine metabolism by arginase and NOS can be measured in the mouse using multitracer stable isotope methods. While NOS activity could be accurately calculated in both models, the isolated lung perfusion model allowed for a more precise calculation of arginase activity compared to the infusion model. Infection of the lung with* Pseudomonas* results in increased NOS and arginase activity in lung and isolated increases of arginase in other organs including trachea and liver. This suggests that infection-induced changes in pulmonary L-arginine metabolism lead to an imbalance of NOS and arginase not only in the lung but also in other organ systems. Whether this imbalance in L-arginine metabolism results in systemic regulatory responses to control overshooting NOS activity or contributes to associated complications such as inflammation and airway narrowing or remodeling, as suggested for asthma [33–35], needs to be further investigated. Further studies are needed to show whether multitracer stable isotope techniques could be utilized to accurately assess the L-arginine metabolism beyond the balance of NOS and arginase.

## Supplementary Material

The supplement that will be available online is a detailed description of specific methods used. The abbreviated version of these methods is already provided in the manuscript in the Methods section in paragraph style.

## Figures and Tables

**Table 1:** Isotope concentrations used for primed constant infusion.

Isotopes	Prime [nmol/0.25 mL] in 20 s	Constant infusion [nmol/mL/h] for 45 min
L-arginine (*m* + 6)	850	1700
L-ornithine (*m* + 2)	425	850
L-citrulline (*m* + 5)	215	430

**Table 2:** Tissue L-arginine metabolizing enzyme activities after stable isotope infusion *invivo*.

	NOS (nmol/g mouse/h)			Arginase (MPE)		
	Control	Infection	*P* value	Control	Infection	*P* value
Lung	9.0 ± 1.4	254.6 ± 36.3	0.0006	16.8 ± 1.6	23.4 ± 1.9	0.017
Trachea	8.6 ± 1.7	6.5 ± 1.3	0.362	17.2 ± 2.0	26.5 ± 3.3	0.043
Liver	1478 ± 294	2208 ± 417	0.136	7.8 ± 0.6	14.7 ± 1.5	0.001
Kidney	16.6 ± 3.6	19.9 ± 1.9	0.231	26.9 ± 3.4	19.0 ± 3.1	0.244
