# Supplementary material for: Multitracer Stable Isotope Quantification of Arginase and Nitric Oxide Synthase Activity in a Mouse Model of Pseudomonas Lung Infection
Source: Mediators Inflamm. 2014 Aug 11;2014:323526. doi: 10.1155/2014/323526 (PMC4142665; doi:10.1155/2014/323526)
Supplement: Supplementary file 1 — The supplement that will be available online is a detailed description of specific methods used. The abbreviated version of these methods is already provided in the manuscript in the Methods section in paragraph style. [file 323526.f1.doc]

**S U P P L E M E N T – Detailed methods for multi-tracer studies**

**Constant tracer infusions**

All mice received a primed (nmol) and constant infusion (nmol/h) of three stable isotopes: L-arginine.HCl [U-^13^C_6_, 97-99%] (prime: 850, constant: 1700), L-citrulline [5-^13^C, 99%: 4,4,5,5,-D_4_, 93%] (prime: 425, constant: 850) and L-ornithine.HCl [^15^N_2_, 98%] (prime: 215, constant: 430), (Cambridge Isotope Laboratories Andover, MA) given into the jugular vein of the mouse. The solution was infused at a rate of 1mL/h and isotopic steady state was achieved within 30 min (Figure 1). Blood was collected via the tail vein at baseline (before infusion) and a plateau sample was collected at 45 min which was later processed for plasma by centrifugation. The mice were then euthanized with ketamine/xylazine and necropsy was performed to isolate the lung and trachea for subsequent mass spectrometric (MS) analysis.


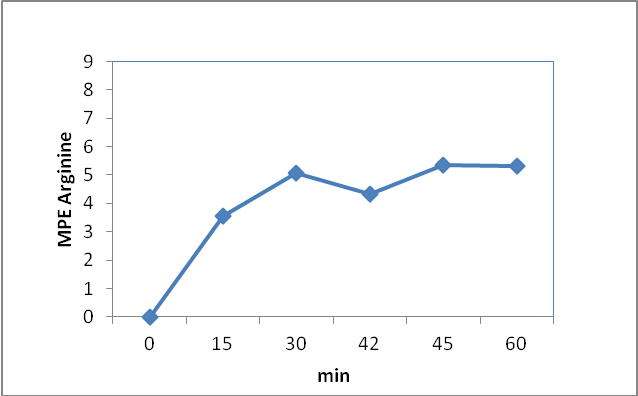


**Figure 1:** Arginine enrichment in plasma after prime-constant infusion

**Analytical procedures**

Tissues (lung and trachea) were homogenized in 2.5 mL, 0.1% formic acid (FA) and 11.25 mL methanol (MeOH) per gram tissue. After centrifugation for done for 10 min at 13,000 rpm and the supernatants were stored at -80^o^C.

**Preparation for tandem mass spectrometry**

*Plasma.* 50 µL plasma was deproteinated with 500 µL of methanol. After centrifugation the supernatant was dried under nitrogen (N_2_). The dried amino acids were reconstituted in 250 µL, 10mM ammonium acetate (NH_4_Ac), pH= 4.1 for MS analysis.

*Tissue methanol supernatant extract.* 250µL of the frozen tissue supernatant was also dried under N_2_. The dried amino acids were reconstituted in 10mM NH_4_Ac (pH= 4.1); 250 µL for lung tissue, and 150 µL for trachea.

**LC-MS/MS analysis**

An API 4000 triple quadrupole mass spectrometer (Applied Biosystems/MDS SCIEX) operated in positive ionization mode with the TurboIonSpray ionization probe source (operated at 5,800 V and 600^o^C), coupled to an Agilent 1100 HPLC system (Agilent Technologies Canada, Mississauga, ON, Canada) was used as previously described . All aspects of data acquisition were controlled using The Analyst NT v. 1.4.1 software.

As the D-isomers of the amino acids were high in plasma and even higher in the tissues, the L- and the D- forms were separated using a chiral Chirobiotic T (teicoplanin) HPLC column, 25cm x 4.6 mm, 5 um (Astec, Whippany, NJ, USA) using an isocratic gradient with 95:5 (10mM NH_4_Ac, pH=4.1) : (2:1 MeOH: 0.1% FA in acetonitrile (ACN) buffer at 700 µl/min, as previously reported . The injection volume was 20 µL with an overall analysis time (injection-to-injection) of 35 min. The retention times of L- and D-arginine were 24.7 and 28.8 min, respectively, for L- and D-citrulline were 5.7 and 6.5 min, respectively, and for L- and D-ornithine were 15.8 and 16.6 min, respectively. Maximum sensitivity for L- and D- arginine, citrulline and ornithine was achieved by measuring product ions multiple reaction monitoring from the fragmentation of the protonated [M + H]^+^ molecules of each amino acid. A representative chromatogram of a multitracer study is shown in Figure 2. Only the enrichments of the L-isoforms of the amino acids were analyzed and are presented for the study.

**Figure 2:** Representative chromatogram of a multi-tracer study

The [M + H]^+^ precursor (parent) ion for each of the unlabeled amino acids was (m/z 175.1 for arginine, 176.1 for citrulline and 133.1 for ornithine). The signal for the most abundant product (daughter) ions (m/z 70.1 for arginine, citrulline and ornithine were also optimized.

The labelled L-arginine.HCl [U-^13^C_6_, 97-99%] was identified using the parent/daughter ion transtion m/z 181.2/74 and was used to calculate arginine flux. The L-citrulline [5-^13^C, 99%: 4,4,5,5,-D_4_, 93%] was used to calculate the citrulline flux which was identified using parent/daughter pair of 181.1/75. The L-ornithine.HCl [^15^N_2_, 98%] was used to calculate the ornithine flux which was identified using parent/daughter pair of 135.2/71 .

To measure NOS activity, the transfer of label from arginine to citrulline was identified using parent/daughter pair of 182.1/74. To measure arginase activity, the transfer of label from arginine to ornithine was identified using parent/daughter pair of 138.2/75.

**Table 1:** Parent/daughter pairs of the amino acids used for mass spectrometry

| Isomers Used | Parent (m/z) | Daughter (m/z) |
| --- | --- | --- |
| **Arginine** |  |  |
| Unlabelled | 175.2 | 70 |
| U-^13^C_6_ | 181.2 | 74 |
| **Citrulline** |  |  |
| Unlabelled | 176.1 | 70 |
| 5-^13^C | 181.1 | 75 |
| From Arg- U-^13^C_6_ | 182.1 | 74 |
| **Ornithine** |  |  |
| Unlabelled | 133.2 | 70 |
| ^15^N_2_ | 135.2 | 71 |
| From Arg- U-^13^C_6_ | 138.2 | 74 |

**Calculations**

Previously published equations were used to calculate the L-arginine metabolism in blood and tissues . The isotopic enrichment was expressed as mole percent excess (MPE), calculated as enrichment at plateau minus the background measurement at baseline . The enrichment values were then used to calculate the whole body flux (Q) for each of the infused amino acids using the following formula :

Q (µmol/kg/h) = isotope infusion rate x [(enrichment_isotope_/enrichment_plasma/tissue_)-1]

The measurement of NOS activity was derived from the conversion of infused arginine to citrulline using the following formula:

NOS Activity = (Enrichment of Arg>Cit/Enrichment of Arg) x Q_cit_

The measurement of Arginase activity was determined in a similar fashion from the conversion of infused arginine to ornithine using the following formula:

Arginase Activity = (Enrichment of Arg>Orn/Enrichment of Arg) x Q_orn_

**References**

[1] K.L. Urschel, M. Rafii, P.B. Pencharz, R.O. Ball, A multitracer stable isotope quantification of the effects of arginine intake on whole body arginine metabolism in neonatal piglets, Am J Physiol Endocrinol Metab 293 (2007) E811-8.

[2] C. Tomlinson, M. Rafii, R.O. Ball, P. Pencharz, The significance of d-isomers in stable isotope studies in humans is dependent on the age of the subject and the amino acid tracer, Metabolism 59 (2010) 14-19.

[3] C. Tomlinson, M. Rafii, R.O. Ball, P. Pencharz, Arginine synthesis from enteral glutamine in healthy adults in the fed state, American Journal of Physiology-Endocrinology And Metabolism 301 (2011) E267-E273.

[4] D.C. Wilson, M. Rafii, R.O. Ball, P.B. Pencharz, Threonine requirement of young men determined by indicator amino acid oxidation with use of L-[1-13C] phenylalanine, The American Journal of Clinical Nutrition 71 (2000) 757-764.
